# Study on Porosity in Zinc Oxide Ultrathin Films from Three-Step MLD Zn-Hybrid Polymers

**DOI:** 10.3390/ma14061418

**Published:** 2021-03-15

**Authors:** Richard Berger, Martin Seiler, Alberto Perrotta, Anna Maria Coclite

**Affiliations:** Institute for Solid State Physics, Graz University of Technology, NAWI Graz, Petersgasse 16, 8010 Graz, Austria; richard.berger@tugraz.at (R.B.); martinseiler44@gmail.com (M.S.); alberto.perrotta@cnr.it (A.P.)

**Keywords:** molecular layer deposition, porosimetric ellipsometry, porous materials, zinc oxide

## Abstract

Deriving mesoporous ZnO from calcinated, molecular layer deposited (MLD) metal-organic hybrid thin films offers various advantages, e.g., tunable crystallinity and porosity, as well as great film conformality and thickness control. However, such methods have barely been investigated. In this contribution, zinc-organic hybrid layers were for the first time formed via a three-step MLD sequence, using diethylzinc, ethanolamine, and maleic anhydride. These zinc-organic hybrid films were then calcinated with the aim of enhancing the porosity of the obtained ZnO films. The saturation curves for the three-step MLD process were measured, showing a growth rate of 4.4 ± 0.2 Å/cycle. After initial degradation, the zinc-organic layers were found to be stable in ambient air. The transformation behavior of the zinc-organic layers, i.e., the evolution of the film thickness and refractive index as well as the pore formation upon heating to 400, 500, and 600 °C were investigated with the help of spectroscopic ellipsometry and ellipsometric porosimetry. The calculated pore size distribution showed open porosity values of 25%, for the sample calcinated at 400 °C. The corresponding expectation value for the pore radius obtained from this distribution was 2.8 nm.

## 1. Introduction

Porous ZnO thin films can be of interest for a broad range of applications, e.g., as gas sensors or for photocatalytic degradation of dyes, as increasing the active surface area of the films enhances some of their functional properties, e.g., adsorption capacity [1]. Our group has recently shown that hybrid Zn-based films, obtained via both unsaturated plasma-enhanced atomic layer deposition (PE-ALD) and molecular layer deposition (MLD), followed by calcination, offer the possibility to obtain micro- and mesoporous ZnO thin films with precise thickness control [2,3]. Especially with MLD-derived porous ZnO, a high pore content was achieved. First, a metal-organic hybrid “zincone” thin film was deposited using diethyl zinc (DEZ) and ethylene glycol (EG) as precursors. Then, the organic content was calcinated by heating the film up to 600 °C in air. With this process, we obtained a max open porosity of 19.3% with a mean pore radius of 2.3 nm. 

The aim of this work was to obtain ZnO with a higher pore content, and we explored the possibility of increasing the organic component of the hybrid polymer by combining different organic precursors with the DEZ. In this context, MLD allows thin films to obtain peculiar properties, above all the high control on the film composition (compared, for example, with chemical vapor deposition of metal-organic compounds) and the (sub)-nm thickness control [4,5].

**Figure 1:**
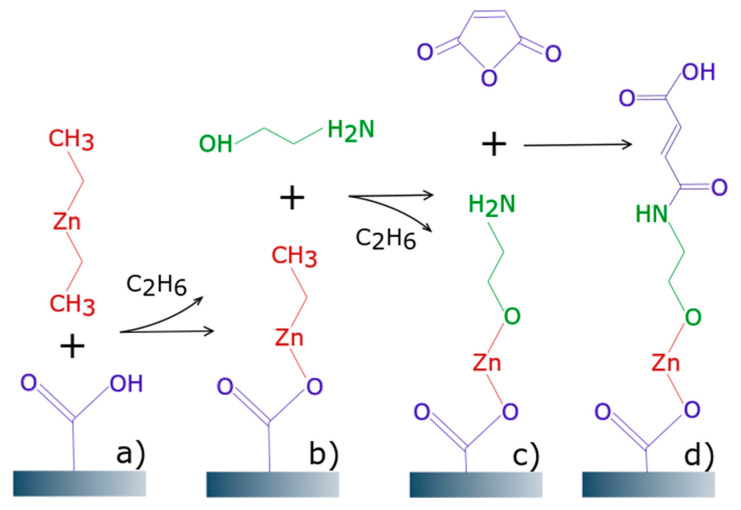
MLD (molecular layer deposited) reaction scheme: (**a**) Diethyl zinc (DEZ) reacting with surface hydroxyl group coming from the previous cycle; (**b**) ethanolamine (EA) reacting with ethyl surface group; (**c**) maleic anhydride (MA) ring-opening reaction; (**d**) monolayer formed after one MLD cycle.

While the formation of zinc-organic films in a two-step MLD deposition process (often called AB sequence) is very well known [4,6,7] (especially for the precursors DEZ and EG), zinc-organic hybrid films using more organic precursors within a single multistep deposition process have been successfully shown only for the aluminum metallic precursor [8]. The growth of metal-organic polymer films with the help of a three-step reaction sequence (which can be referred as ABC sequence) was investigated using the DEZ as the zinc precursor, and maleic anhydride (MA) as well as ethanolamine (EA) as the organic precursors. The proposed reaction scheme for the ABC cycle using DEZ, MA, and EA is illustrated in Figure 1.

The effects of a variable organic content within the zinc-organic polymer, provided by different organic precursors, on the porosity of the calcinated film could be observed by ellipsometric porosimetry (EP). With this method, we aim at answering the question if a larger organic content with a linear chain structure within the hybrid film would influence the pore size distribution of the zinc oxide film obtained upon calcination.

The zinc-organic polymer films produced via sequential molecular layer deposition of DEZ, EA, and MA were calcinated by heating the samples up to 600 °C in air. During the heating of the metal-organic hybrid films, in situ ellipsometry measurements were carried out measuring both thickness and refractive index. Thus, detailed analysis of the film’s structural transformation during the temperature increase could be performed.

In order to investigate the porosity properties of the calcinated ZnO thin films, EP measurements were performed for samples heated to both 400 °C and 600 °C. This allowed for a direct comparison to samples produced via a simple two-step sequence using DEZ and EG, calcinated at the same heating temperatures [2]. Besides the total fraction of open porosity (describing the fraction of voids accessible from the surface of the sample), a pore size distribution (PSD) was derived from the data measured via EP.

## 2. Experimental

### 2.1. MLD Setup

**Figure 2:**
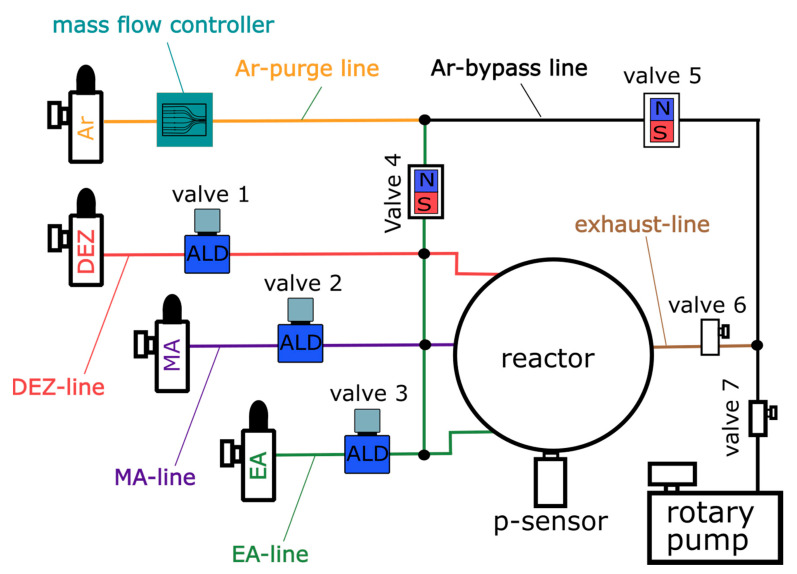
MLD setup scheme: Valve 1–3: ALD valves for precursor inlet control, valve 4–5: Magnetic valves for switching the Ar-flow, mass flow controller for setting 16 sccm Ar-flow, valve 6–7: Manual valves to de-/couple system and vacuum pump.

The MLD setup, previously described [2,3], was modified in order to accommodate three precursors: DEZ, EG, and MA (all obtained from Sigma Aldrich, St. Louis, MO, USA). The MLD setup scheme is illustrated in Figure 2. The thin films were deposited on single-side polished c-Si (100) substrates (Siegert Wafer, Aachen, Germany). An in-house programmed software [9] was used for automating the deposition process, allowing the precise control of the ALD valves (Swagelok ALD3, Solon, OH, USA) employed to define the precursor exposure and purging times during the MLD process (see Figure 2: Valve 1–3). During the deposition, a simple rotary vane pump (RVP, Pfeiffer DUO 005 M, Aßlar, Germany) was used for evacuation, while Ar was constantly flown into the system using a mass flow controller (MKS MF1-C, Andover, MA, USA) driven by the automation platform (MKS PAC 1000, Andover, MA, USA). Using an Ar flow rate of 16 sccm resulted in a process pressure of 0.5 Torr. A special attention was paid to a proper heating of the whole setup in order to avoid condensation of the precursor vapors inside the reaction chamber, which would cause unwanted chemical vapor deposition (CVD)-like growth, resulting from the reactions of the different precursors in the gas phase and subsequent deposition onto the substrate. The system temperatures are listed in Table 1. The relatively low temperature of the sample stage (60 °C) was chosen to favor the adsorption of the organic precursors, especially EA.

### 2.2. Thin-Film Characterization: Spectroscopic Ellipsometry

The thin films were investigated with the help of spectroscopic ellipsometry (SE, J.A. Woollam M-2000V, Lincoln, NE, USA) determining the thickness and refractive index of the films [10]. The measured data were evaluated with the software CompleteEASE^®^ (Version 5.19, 2018, J.A. Woollam, Lincoln, NE, USA) using a three-layer model taking into account the Si substrate, a SiO_2_ native oxide layer, and the actual thin film. For fitting the optical properties of the thin film to the measured data a Cauchy model [10] (Equation (1)) was employed.

(1) $$n=A+\frac{B}{\lambda^{2}}+\frac{C}{\lambda^{4}}$$

The Cauchy model describes the real part of the refractive index *n* (hereafter, we will only refer to the real part when talking about the refractive index) as a function of the wavelength *λ* and the fitting parameters *A*, *B*, and *C*. The ellipsometry data were fitted in a spectral range between 400–1000 nm excluding wavelengths in the range of the ZnO bandgap (3.3 eV ≙ 376 nm). This offers the advantage that absorption effects are negligible when fitting both metal-organic hybrid and crystalline ZnO films. This approach simplifies the further treatment of the data since the imaginary part of the refractive index does not need to be taken into account. This approach allows the measurement of the total film thickness and therefore the growth per cycle (GPC) of the metal-organic thin film by dividing the overall thickness by the number of MLD cycles. Furthermore, the behavior of the metal-organic film upon heating can be investigated by measuring the film thickness as well as the refractive index in situ as a continuous function of temperature while heating and cooling the samples.

In the latter, the refractive index measured via SE was used to make qualitative statements on the density of the measured films. Therefore, the refractive index was described via the Lorentz oscillator model for only electronic excitation of the measured film. This is a reasonable assumption since the resonance frequencies of ionic and orientation polarization are much lower than the spectrum used in SE. In case of a transparent, non-magnetic film, the Lorentz model gives Equation (2).

(2) $$n=\sqrt{1+\frac{\omega_{p,e}^{2}}{\omega_{0,e}^{2}-\omega^{2}}}$$

(3) $$\omega_{0,e}^{2}=\frac{K_{e}}{m_{e}},\;\omega_{p,e}=\frac{e^{2}N_{e}}{\varepsilon_{0}m_{e}}$$

Equation (2) describes the refractive index in terms of the plasma frequency *ω_p_*_,*e*_, and resonance frequency *ω*_0,*e*_ (Equation (3)) for electronic polarization of the material. Since *ω*_0_, e is usually in the UV regime, one can further approximate *ω*_0,*e*2_ ≫ *ω*_2_ in case of SE, yielding Equation (5).

(4) $$n=\sqrt{1+\frac{e^{2}N_{e}}{\varepsilon_{0}K_{e}}\;}$$

Equation (4) describes the refractive index of a non-magnetic, transparent material in the visible spectrum as a function of only the average electron density *N_e_* and the mean “spring constant” *K_e_*. *K_e_* (see Equation (3)) is given by the spatial curvature of the bond potential of the electrons around their equilibrium position. Despite the fact that Equation (4) uses an extremely simplified model, it allows for an easy qualitative description of a material in terms of its electron, and therefore mass, density and the binding structure of electrons.

### 2.3. Porosity Measurements: Ellipsometric Porosimetry

A quick and simple yet accurate way to determine the amount of open porosity of a thin film is via ellipsometric porosimetry (EP) [11]. EP consists in measuring the refractive index of a sample via SE, as described in [2], while simultaneously increasing the partial pressure of a probing vapor, in this case water, in the atmosphere surrounding the sample. Successively, the probing molecules condense inside the pores of the thin film, increasing its overall refractive index. Once all pores are filled with the condensate (also called probe), one can calculate back the volume fraction of the pores inside the film via the Lorentz-Lorenz effective medium approximation [12], yielding Equation (5).

(5) $$\frac{V_{probe}}{V}=\frac{\frac{n^{2}-1}{n^{2}+2}-\frac{n_{0}{}^{2}-1}{n_{0}{}^{2}+2}}{\frac{n_{probe}{}^{2}-1}{n_{probe}{}^{2}+2}}$$

*V_probe_* describes the total volume of condensate inside all pores while *V* is the overall film thickness. Hence, Equation (5) relates the volume fraction *V_probe_*/*V* of pores that are filled with condensate to only measurable quantities such as the refractive index of the film before being exposed to any probing vapor (*n*_0_), the refractive index of the film containing the condensate (*n*) and the well-known refractive index of the probing gas in the liquid state (*n_probe_*).

Besides the quantification of the pore fraction within the thin film, EP provides the possibility to obtain a pore size distribution [13] (PSD), in the mesoporous regime, by means of the Kelvin equation (Equation (6)).

(6) $$\frac{1}{r_{1}}+\frac{1}{r_{2}}=\frac{RT}{\gamma V_{m}}*ln\left(\frac{p}{p_{0}}\right)$$

**Figure 3:**
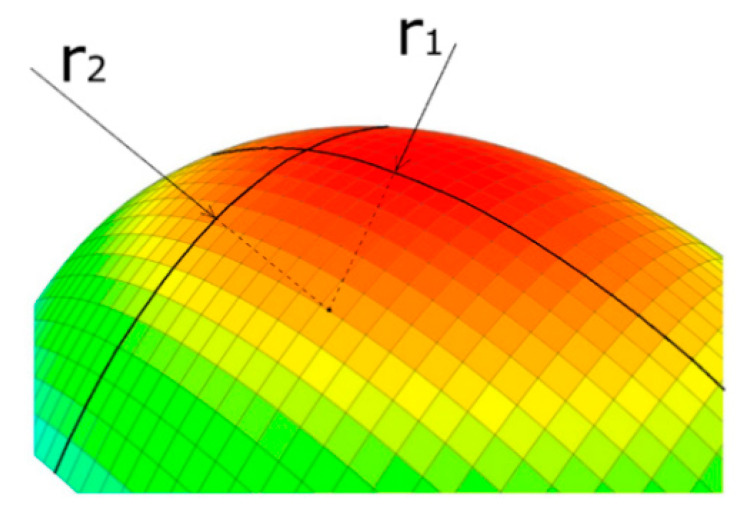
Liquid surface with two perpendicular surface trajectories of curvature *r*_1_ and *r*_2_.

Equation (6) shows the Kelvin equation in its most general form, relating the vapor pressure of a liquid (*p*) to its surface topography, described by the radii, *r*_1_, and *r*_2_, related to the local curvature of two perpendicular surface trajectories (see Figure 3). *R* is the universal gas constant, *T* the temperature, *V_m_* the molar mass, *γ* the surface tension, and *p*_0_ the vapor pressure of the liquid in case of a flat surface, i.e., the saturation vapor pressure.

**Figure 4:**
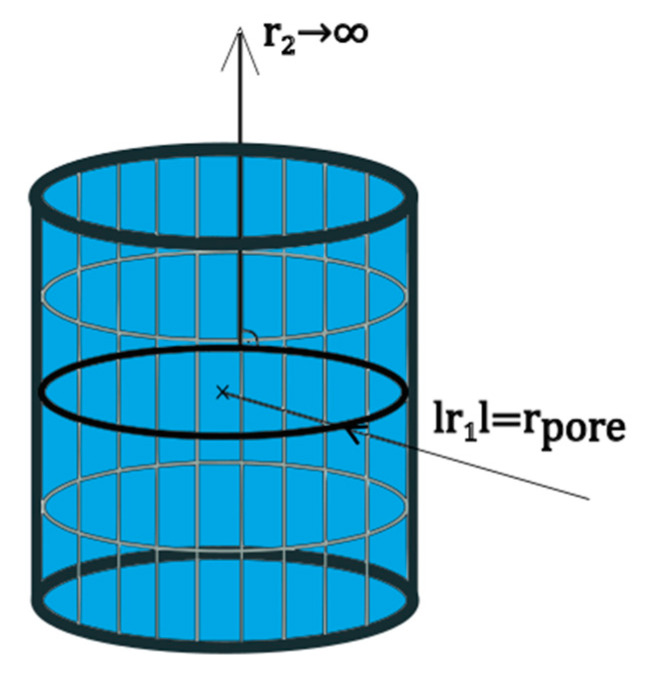
Liquid layer inside cylindrical pore.

Deriving a PSD from the general form of the Kelvin equation (Equation (6)) requires a model of the geometrical shape of the pores as well as an assumption that relates the shape of the pores to the shape of the condensate surface. For a simple mathematical treatment, a cylindrical model was chosen for the pore shape. In the adsorption regime, assuming a thin layer of condensate, the shape of the liquid surface is given by the shape of the pores (see Figure 4). This assumption is not true in the desorption regime since in that case the liquid-gas interface is more complex and requires knowledge about the contact angle between the condensate and the porous material in order to justify any assumption on the liquid-gas interface topography.

Applying the assumption of a cylindrical concave condensate surface to Equation (6) yields *r*_1_ → ∞, *r*_2_ → −*r_pore_* (see Figure 4), leading to the Kelvin equation for cylindrical pores in the adsorption regime (Equation (7)).

(7) $$r_{pore}=\frac{\gamma *V_{M}}{R*T*\;ln\left(\frac{p_{0}}{p}\right)}$$

Equation (7) describes the maximum radius of pores in which the probing gas can condense at a given partial pressure *p*.

In case of water serving as the probe, *p*/*p*_0_ is simply given by the relative humidity RH, which can easily be measured. In order to measure the PSD, the RH in the atmosphere surrounding the sample is successively increased and measured from 0 to 100% while simultaneously measuring the refractive index *n* of the sample. Combining Equations (5) and (7) one can obtain the volume fraction of pores filled with water as a function of the maximum pore radius. This, in turn, is equivalent to a cumulative distribution of the pores with respect to their radius. Hence, the PSD can simply be calculated by taking the derivative of this cumulative function with respect to the pore radius and subsequent normalization of the distribution. 

**Figure 5:**
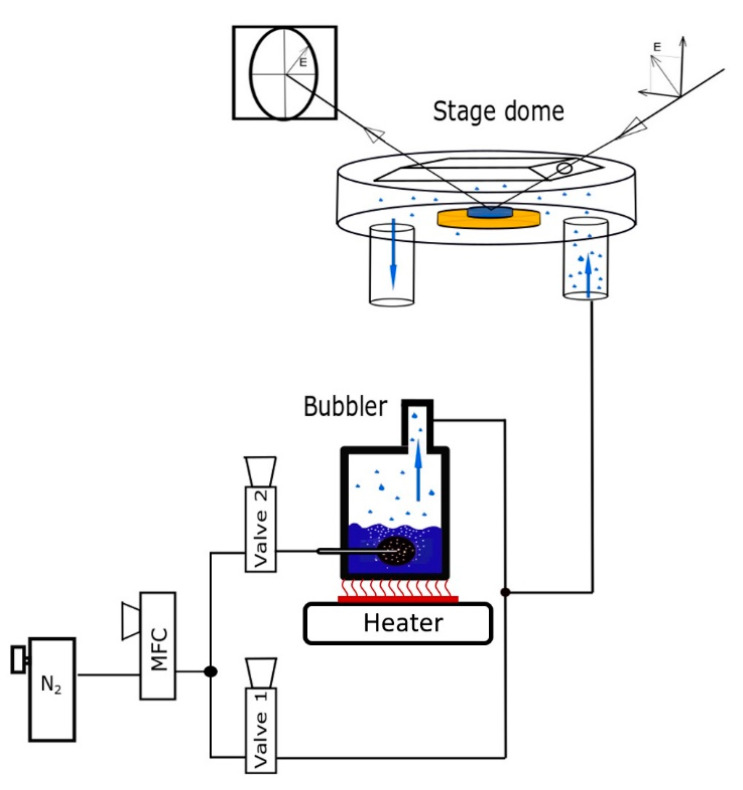
Experimental setup for ellipsometric porosimetry (EP) measurements: The opening ratio between valve 1 and valve 2 sets the relative humidity in the sample stage dome (sample in blue in the cartoon). Additional heating increases the RH beyond 85%. The refractive index of the sample inside the dome is measured via ellipsometry. A RH-sensor measures the humidity inside the dome. The water droplet produced in the bubbler and reaching the stage dome are represented as blue dots.

The experimental setup for the EP measurements is illustrated in Figure 5. To vary the relative humidity in the atmosphere surrounding the samples to be measured, nitrogen is flown through a custom-made water bubbler into a dome surrounding the sample. By varying the opening ratio between valve 1 and valve 2, the amount of nitrogen going through the bubbler/bypass can be adjusted changing the RH inside the sample dome. Using this procedure, a maximum RH of 85% can be reached inside the dome. For further increase, the bubbler must be heated. In a typical EP experiment, the RH humidity is increased from 0 to 100% and measured with a sensor inside the sample dome. Simultaneously, the changing refractive index of the sample is measured via ellipsometry thanks to two transparent windows in the sample dome.

## 3. Results

### 3.1. Saturation Curves

**Figure 6:**
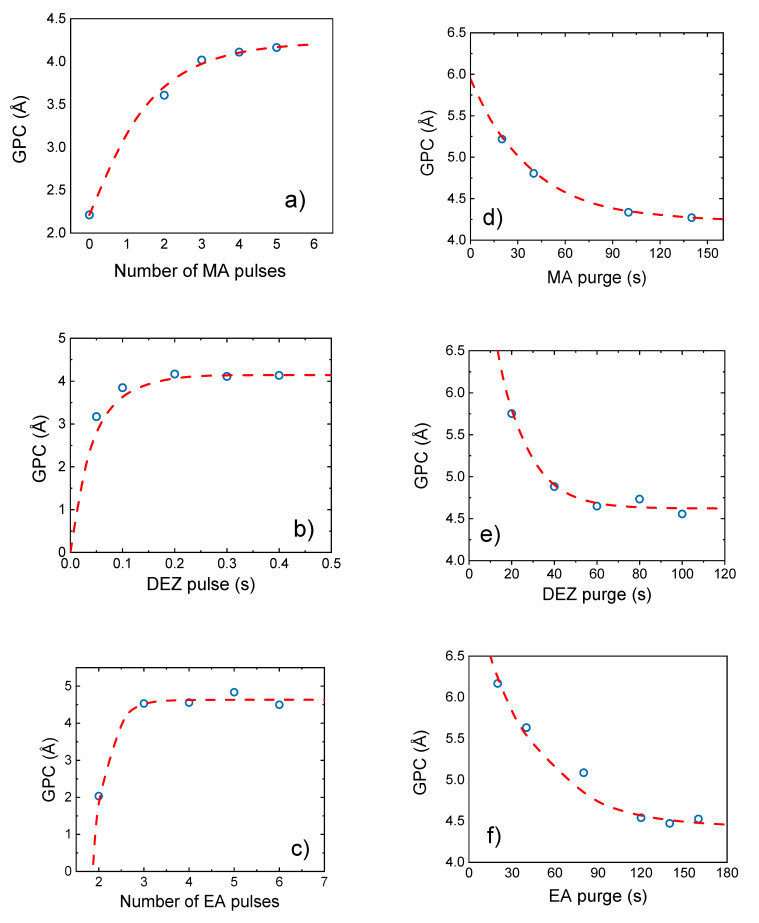
Saturation curves for the 3-step ABC cycle using the precursors DEZ, EA, and MA and a stage temperature of 60 °C. The growth per cycle (GPC) is reported as a function of (**a**) variable number of MA pulses (0.1 s pulse time) during one MA exposure step. (**b**) Single DEZ pulses of variable pulse time. (**c**) Variable number of EA pulses (1.5 s pulse time) during one EA exposure step. (**d**–**f**) MA, DEZ, and EA purging using an Ar flow rate of 16 sccm.

The deposition of the zinc-organic thin films, starting from the precursors DEZ, EA, and MA was first optimized in order to guarantee a self-limiting MLD process with limited CVD-like growth. For this, the characteristic saturation curves for the three-step ABC MLD cycle were measured and are reported in Figure 6.

The saturation curves display the GPC as a function of the precursor exposure and purging times (*t_exp_* and *t_purge_*, respectively). The measured data points for the purging and precursor exposure times were fitted with an exponential decay and saturation-type exponential functions, respectively. This allowed the extrapolation of the GPC in the saturation regime of each curve as the limit of the according function. In case of EA and MA, multiple shorter pulses, respectively with a pulse time of 1.5 s and 0.1 s, were used in a single precursor exposure step (see Figure 6). A delay time of 10 s between the single short pulses was chosen. This approach simply allowed for a more precise control of the precursor dosing compared to a very long single exposure step. During longer exposure steps the pressure inside the precursor vessels falls far below the desired vapor pressure, complicating the exact control of the precursor dosing. This effect can be avoided with multiple subsequent MA pulses (Figure 6). Within the DEZ exposure step, the duration of one single precursor exposure time was varied. In case of DEZ this was possible since the gas pressure inside the vessel did not decrease significantly during the short single pulses. Interestingly, the saturation curve of the MA number of pulses (Figure 6a) shows a GPC also at 0 pulses, i.e., without MA addition. This is due to the fact that DEZ and EA react sequentially with each other and can form a film already without MA. Hence, in case of a saturated two-step MLD sequence using only DEZ and EA as the deposition precursors Figure 6a shows a GPC of 2.2 Å /cycle. The elimination of any of the other two precursors (DEZ or EA) from the ABC recipe resulted instead in no film growth.

**Figure 7:**
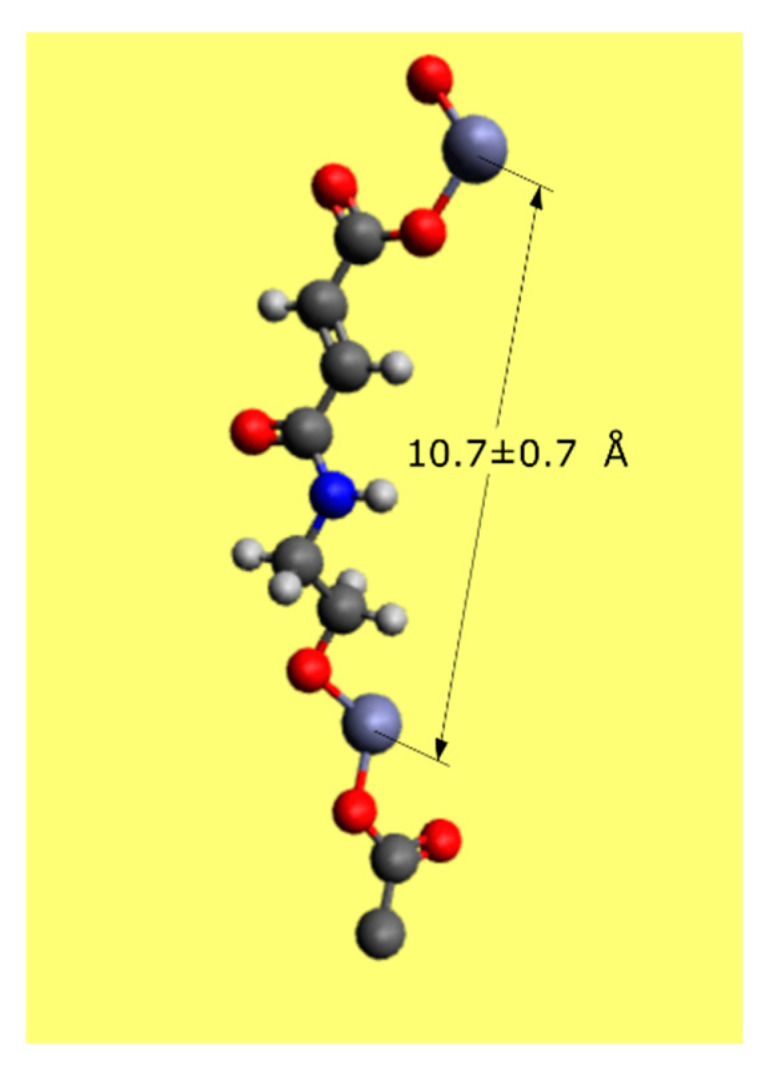
Monolayer molecule geometry simulated via Avogadro yielding a distance of 10.7 ± 0.7 Å between two consecutive Zn atoms.

The ideal deposition recipe was found by choosing the shortest purging and exposure times that are still in the saturation regime. This was 0.3/100/4 × 1.5/140/4 × 0.1/100 s for the DEZ/purge/EA/purge/MA/purge sequence, yielding a saturated GPC of 4.4 ± 0.2 Å /cycle. Little deviations in the saturation value are referred to the fact that the sample thicknesses were measured after short exposure to ambient air (5–20 min), yielding different thickness change due to degradation (discussed later). Still, the maximum deviation for the saturated GPC in all saturation curves lies within 10 %. Such thickness deviations can even be observed for films that have been deposited using the same MLD framework conditions. Here and in the following, the precursor exposure times refer to the opening times of the according ALD-valves (Figure 2, valve 1–3). The observed saturated GPC is shorter than the sum of the nominal length of the precursors, which was determined as 10.7 ± 0.7 Å for a single monolayer molecule using the simulation software Avogadro (see Figure 7). The error for this value was calculated from the distance standard deviation obtained from several simulations converging to slightly different optimized geometries.

A GPC shorter than the nominal molecule length was an observed effect when using linear aliphatic organic precursors. It can be explained by the tilting of the reacted organic species with respect to the sample surface [4,14]. Besides tilting, termination of active surface functional groups reduces the GPC [15]. It is reported that this effect strongly depends on the backbone flexibility of the organic precursor species [15].

For samples that were deposited using the ideal deposition recipe for the DEZ/EA/MA (ABC) sequence, a refractive index of n_@633 nm_ = 1.59 ± 0.01 was measured a few minutes after exposure to air. In the case of samples produced via the DEZ/EG (AB) deposition sequence [2], analogue measurements revealed a refractive index of n_@633 nm_ = 1.55 ± 0.01. To state on the structural difference between the AB/ABC samples, one can exploit Equation (4). Therefore, it is reasonable to introduce the material constant α according to Equation (8):

(8) $$\alpha =\frac{N_{e}}{K_{e}}$$

Acting on Equation (4), one can then calculate *α* for both the AB and ABC samples, yielding the following ratio:

(9) $$\frac{\alpha_{ABC}}{\alpha_{AB}}=1.0$$

Equation (9) suggests that the two film types deposited from both DEZ/EA/MA- and DEZ/EG MLD sequences show similar density and electron bond structure. 

One can use this fact to argue on the possible mechanism of subsurface precursor diffusion as it was observed for ABC type MLD using trimethylaluminum (TMA), EA, and MA [16]. Segethe et al. reported an increase by an order of magnitude for GPC and mass gain per cycle for films deposited from TMA, EA, and MA between 90 °C and 110 °C regarding comparable AB sequence MLD systems. This effect is assigned to the diffusion of TMA into the films during the deposition causing non-self-limiting behavior. In case of DEZ, as the metallic precursor in the ABC sequence, both the GPC and the refractive index (and therefore the mass density) are in the same range as for comparable two-step depositions [2,4,17]. Moreover, Figure 6 shows clear saturation behavior, suggesting a self-limiting deposition process. Therefore, in case of depositions performed using the optimized conditions, we can exclude CVD-like reactions caused by the diffusion of DEZ inside the film. This can be justified in several ways. First of all, Segethe et al. report exposure times of 6 s for all precursors. We instead use multiple shorter pulses in case of the organic precursors and a very short single pulse (0.3 s) in case of DEZ. This leaves less time for precursor diffusion within the film. Furthermore, since diffusion is a temperature driven process, it can be assumed that the lower deposition temperature of 60 °C brings an advantage when it comes to avoiding this side effect in the MLD deposition.

**Figure 8:**
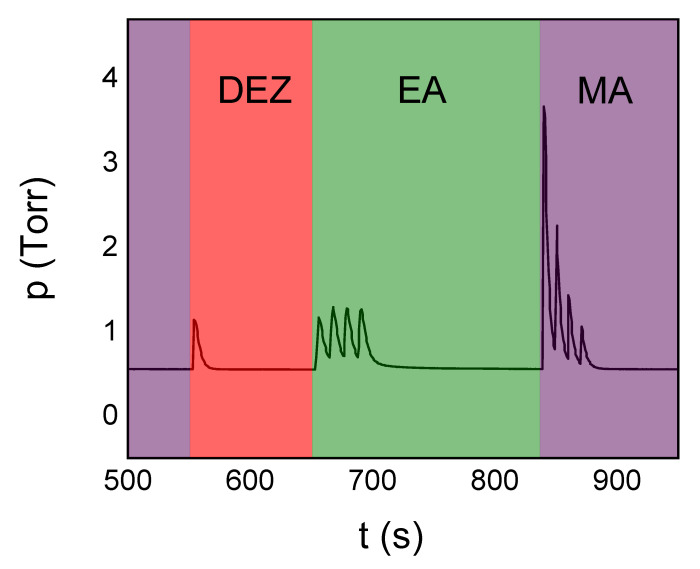
Pressure values as read during the precursor pulses in a typical ABC recipe.

The partial pressure increase for the precursor exposure steps using the ideal deposition recipe is illustrated in Figure 8. The partial pressure increase was measured as the difference between the pulse peak and the initial pressure value at the beginning of the pulse (which is not necessarily the process pressure in case of subsequent pulses). The single 0.3 s DEZ pulse causes a partial pressure increase of Δp = 0.57 ± 0.01 Torr. Both the EA and MA pulses are separated by a delay time of 10 s. The 1.5 s EA pulses cause a constant pressure increase of Δp = 0.58 ± 0.02 Torr, whereas the 0.1 s MA pulses successively decrease in magnitude from Δp = 3.2 ± 0.1 Torr for the first pulse to Δp = 0.43 ± 0.04 Torr for the fourth pulse. The exponential decay time constants of the DEZ/EA/MA pulses are 4.2 ± 0.4 s, 3.4 ± 0.3 s, and 9 ± 1 s, respectively. Despite the comparably short exponential pulse decay times, the purging times for saturated self-limiting MLD growth are 100/140/100 s after the DEZ/EA/MA exposure.

In case of comparable AB cycle MLD processes, depending on the exact precursor type and experimental setup, the purging times are in the range between 30 and 60 s [2,6,18,19]. The purging times for AB cycle MLD at the same setup were 60 s after both DEZ and EG. This is significantly less than in case of the DEZ/EA/MA sequence. In case of simple AB sequence MLD, purging causes the removal of physiosorbed reactants [6]. However, in case of ABC cycle MLD deposition of alucones higher purging times from 120 up to 300 s are reported [8,16], which is ascribed to the subsurface diffusion of TMA inside the film according to Segethe et al. Therefore, it is highly likely that also in the case of the DEZ/EA/MA deposition sequence the precursors can diffuse inside the growing film and require long purging times to be removed. This is in line with the comparably fast exponential decay of the pressure pulses. Those reactants diffusing within the film are not causing any significant partial pressure increase within the reactor while still increasing the GPC beyond the saturation value due to reactions which are not restricted to the sample surface explaining the great difference between the exponential pulse decay times and the according purging times for self-limited film growth.

### 3.2. Zinc Organic Hybrid Film Stability

**Figure 9:**
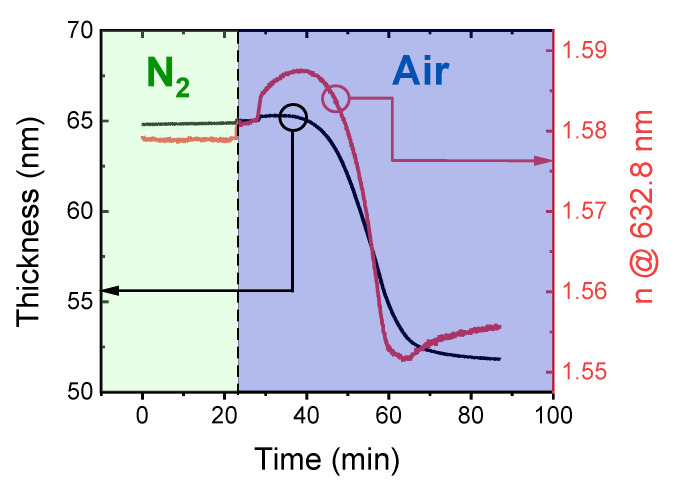
Zinc organic hybrid film degradation: Thickness and refractive index measured upon exposure to nitrogen (t < 22 min) and ambient air (t > 22 min).

It is also worth investigating whether the zinc organic films obtained from ABC cycle MLD were stable upon exposure to the atmosphere. Figure 9 shows the refractive index and the thickness of a zinc organic film upon exposure to both nitrogen and ambient air. When solely exposed to a nitrogen atmosphere, the film shows no significant changes. Yet, after the first 22 min, the film was exposed to ambient air, and it started to degrade quickly. The first step in the refractive index at 22 min is an experimental artifact caused by the removal of the ellipsometer stage dome providing the N_2_ atmosphere (see Figure 9). Within the first 20 min of the exposure to air, the refractive index increases from 1.581 to 1.589. This is assigned to an initial infiltration of water from the ambient air into the film. Subsequently, one can observe a rapid drop of the refractive index. In total, within 65 min of air exposure, the refractive index and the film thickness decreased by 1.5% and 20%, respectively, regarding the initial values. This degradation mechanism is assigned to the hydrolysis caused by water from the ambient air penetrating into the film. The degradation of zinc organic hybrid polymers has previously also been investigated in detail by Peng et al. [6]. The instability in air was referred to the hydrolysis of the film caused by water in the ambient, accessible through the pristine porosity in the MLD layers, demonstrated for both Al- and Zn-based hybrid films [8,20,21]. After 65 min of exposure to air, no further degradation was observed.

### 3.3. Transformation upon Heating

**Figure 10:**
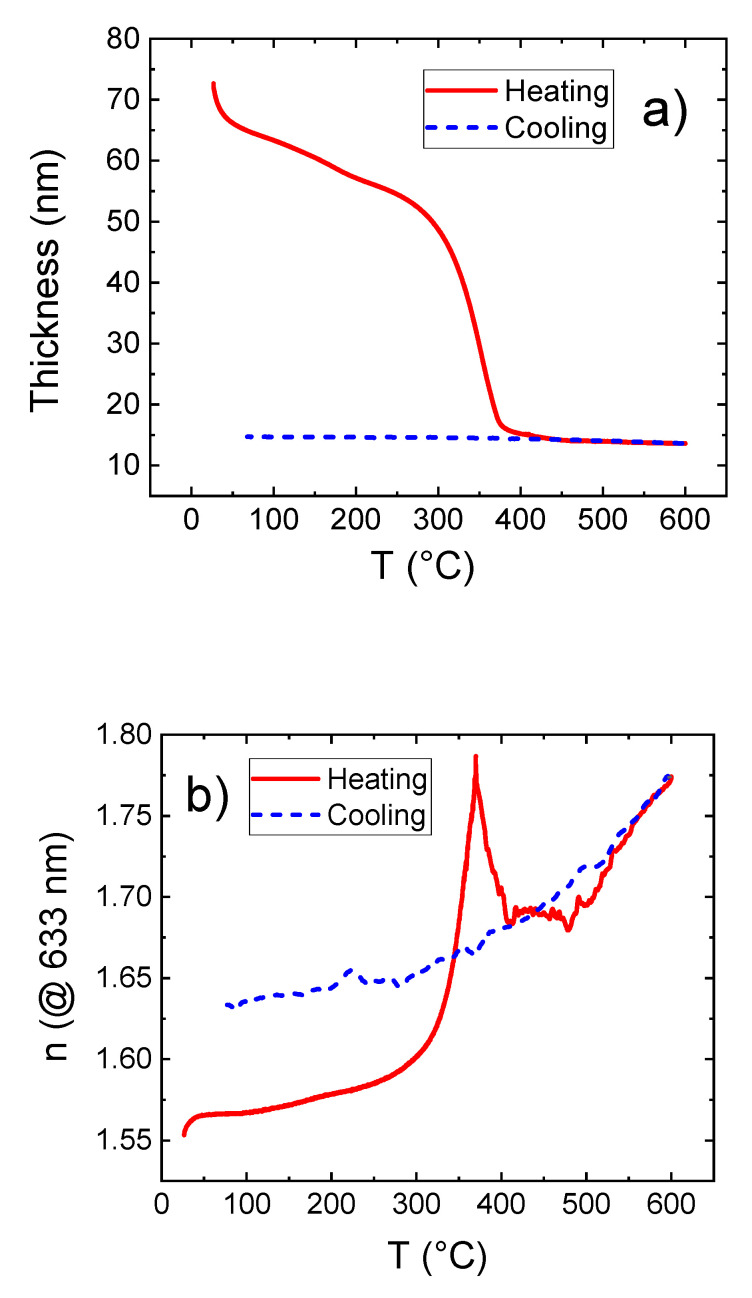
(**a**) Film thickness and (**b**) refractive index as a function of temperature measured during a heating cycle 25 °C–600 °C–25 °C on the zinc-organic film.

To obtain porous zinc oxide, the metal-organic hybrid films were heated from room temperature up to 600 °C and cooled back down to room temperature in ambient air. The heating rate was set to 200 °C/h. The metal-organic samples had been exposed to ambient air for several hours before the heating experiments. The film thickness and refractive index at a wavelength of 633 nm were measured in situ and reported in Figure 10 as a continuous function of the temperature.

It can be observed that below 300 °C, the film thickness decreases moderately with the temperature yielding a total thickness reduction of 35% at 300 °C. However, the refractive index increased by 3% at 300 °C compared to its initial value at room temperature. This indicates that only little organic content is removed within this step. This is in contrast with similar measurements performed on films deposited using only DEZ and EG, which show a rapid thickness drop and a jump of the refractive index already around 110 °C [2].

Figure 10 shows a drastic decrease of the film thickness between 300 °C and 370 °C, along with a rapid increase of the refractive index. This can mainly be assigned to a collapse of the film caused by the removal of organic content together with the starting crystallization of ZnO that is reported around 340 °C [2].

In the range of 370–600 °C, the thickness shows only minor changes while the refractive index undergoes an initial downfall and subsequent increase after a short constant domain. We believe that this behavior is due to a cross effect of collapse/densification and void formation within the film. During the calcination between 270 °C and 370 °C the organic content is not entirely replaced by vacancies in the film. Rather a collapse of the film is observed in this temperature regime. This is reflected in a rapidly decrease in film thickness and an increasing refractive index. Between 370 °C and 400 °C, the organic content is further removed from the film without any further collapse of the film, i.e., the organic content is replaced by voids inside the film. This explains the decreasing refractive index at a constant film thickness. Between 400 °C and 600 °C, the refractive index increases again which can be assigned to an ongoing crystallization of the remaining ZnO at the cost of the film porosity. This crystallization effect has also been observed for films obtained from the AB-cycle deposition process using DEZ and EG as the precursors [2].When the film is cooled back down to room temperature, the thickness stays fairly constant while the refractive index decreases continuously, which is a characteristic behavior of stable crystalline ZnO films [2,22,23,24]. Again, similar behavior has been observed for analogue experiments on zinc-organic samples deposited from DEZ and EG that had successfully been transformed to porous crystalline ZnO by calcination [2].

The final refractive index of the thin film that had been cooled back to room temperature is higher compared to the initial metal-organic hybrid film. This is assigned to the removal of the organic content from the thin film and formation of porosity within the calcination process. The remaining ZnO has a higher mass density and therefore higher electron density which is reflected in an increased index of refraction.

**Figure 11:**
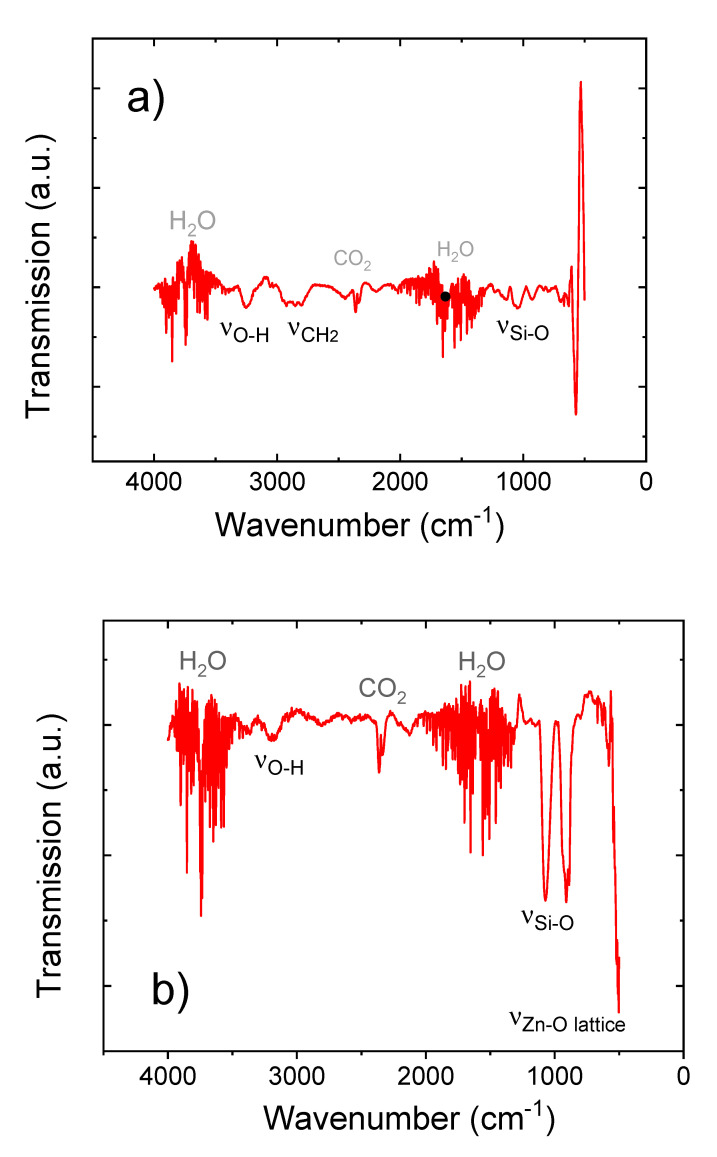
FTIR spectroscopy for samples calcinated at (**a**) 400 °C and (**b**) 600 °C.

Figure 11a,b shows FTIR spectra taken for samples calcinated at both 400 °C and 600 °C, respectively. Both spectra show noisy absorption modes at 3400–4000 cm^−1^ and 1300–2000 cm^−1^ ascribed to remaining water adsorbed inside the porous film, in line with the O-H stretching mode at 3250 cm^−1^. Furthermore, both spectra show typical asymmetric CO_2_ stretching modes at 2362 and 2335 cm^−1^ caused by minor gaseous CO_2_ inside the FTIR spectrometer. In case of the sample calcinated at 400 °C (Figure 11a), another absorption peak can be observed in the range of 2710–3010 cm^−1^ which is ascribed to CH_2_ bending caused by remaining organic content inside the film. This peak is no longer visible for the sample calcinated at 600 °C, indicating the total removal of any organic content from the film. Figure 11b also shows absorption peaks at 1060 and 920 cm^−1^. This could be ascribed to C-O stretching in C-C-O and Zn-O-C moieties [2,6,7], yet it is more likely that these features are due to the oxidation of the Si substrate upon heating. This effect becomes more obvious for the sample heated to 600 °C (Figure 11b), showing clear Si-O stretching modes at 1070 and 910 cm^−1^ [2]. In case of the 600 °C sample, the onset of the characteristic Zn-O stretching mode for crystalline ZnO can be observed below 600 cm^−1^ [2]. Unfortunately, the utilized spectrometer does not cover the regime around 400 cm^−1^, where the Zn-O stretching mode is centered [2].

### 3.4. Porosimetry on Calcinated MLD Zincones

**Figure 12:**
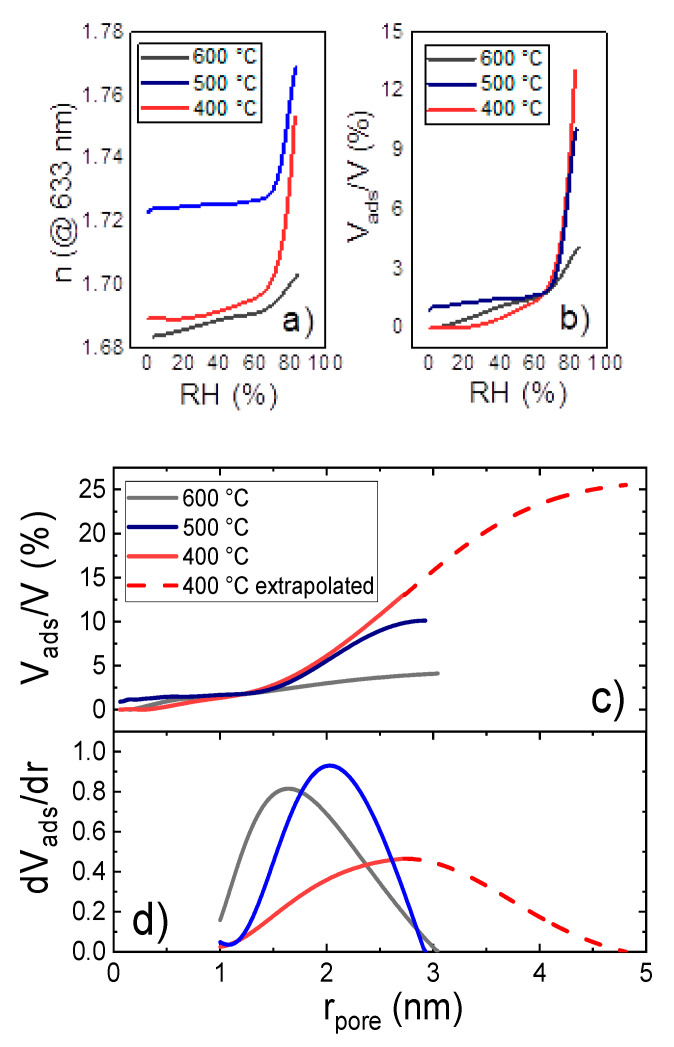
(**a**) Measured refractive index and (**b**) calculated volume fraction of pores filled with condensed adsorbate as a function of the relative humidity. (**c**) Volume fraction of pores filled with condensed adsorbate and (**d**) normalized PSD for pore radii > 1 nm as a function of the pore radius.

EP measurements were performed on thin films that had been produced via the described three-step MLD sequence and subsequentially calcinated by heating various samples to 400, 500, and 600 °C. The related measurements, as well as the resulting PSD, are shown in Figure 12. The refractive index of the film was measured while the RH was increased from 0 to 83 % (Figure 12a).

Applying Equations (5) and (7) to the refractive index variation with RH (Figure 12a), the volume fraction of all pores filled with condensate as a function of the maximum radius of these pores was calculated. Hence, Figure 12c can be interpreted as the cumulative distribution of open pores with respect to the pore radius. Therefore, its derivative yields the according PSD (Figure 12d). Due to limitations in the experimental setup, the RH could not be increased beyond 83%, restricting the detectable pore size to *r_pore_* < 2.8 nm (as it can be calculated from Equation (7)). Figure 12b shows the volume fraction of all pores that are filled with condensate at a specific RH.

Figure 12d shows that the average pore size decreases with the heating temperature: The expectation values for pore radii, calculated from the PSD in the mesoporous regime, are 2.8, 2, and 1.8 nm for the samples that had been heated to 400, 500, and 600 °C, respectively. By comparing the FTIR results with the in situ SE data and EP, at 400 °C there is an increase of the refractive index, pointing out the onset of the crystallization of ZnO, followed by a decrease due to the removal of the organic content in the layer (Figure 10). In the FTIR spectrum, however, the Zn-O stretching is not visible or covered by noise, indicating that the crystallization of ZnO is not as effective at 400 °C as for the AB process. This would account for a higher porosity in the ABC layer, since we demonstrated that for Zn-hybrid layers the crystallization and crystal growth is a competitive mechanism to the pore formation.

The curves of the volume fraction of pores obtained at 400 °C were extrapolated for pore radii bigger than 2.8 nm (RH > 83 %). The measured PSD for the 400 °C sample clearly shows its peak for pores with a radius of 2.8 nm. The PSD for radii > 2.8 nm was assumed to follow a gaussian behavior (dashed red line in Figure 12d). The fast decay of the extrapolated part of the PSD was chosen in order not to overestimate the contribution of bigger pores. This approach was justified by the fact that comparable PSDs show a symmetric or right-skewed behavior, therefore ours is rather an underestimation. The real PSD for the 400 °C sample will most likely have a higher contribution for bigger pores than the extrapolated PSD, meaning that the total porosity is likely to be higher than 25%. This approach also allows the extrapolation of the cumulative distribution (dashed red line in Figure 12c), simply by integrating the (not normalized) extrapolated part of the PSD.

Although the PSD of the 400 °C sample has only been extrapolated, it is obvious that there is a great difference already in the measured datapoints of the PSD comparing the 400 °C to the 500 and 600 °C samples (see Table 2). Moreover, when comparing the 400 °C PSDs of DEZ/EA/MA to the DEZ/EG samples [2], one finds a significant shift of the PSD towards higher pore radii in case of the DEZ/EA/MA samples. This great discrepancy in the shape and center of the PSDs shows the increased mean pore size of the DEZ/EA/MA samples calcinated at 400 °C, despite possible statistical fluctuations.

**Figure 13:**
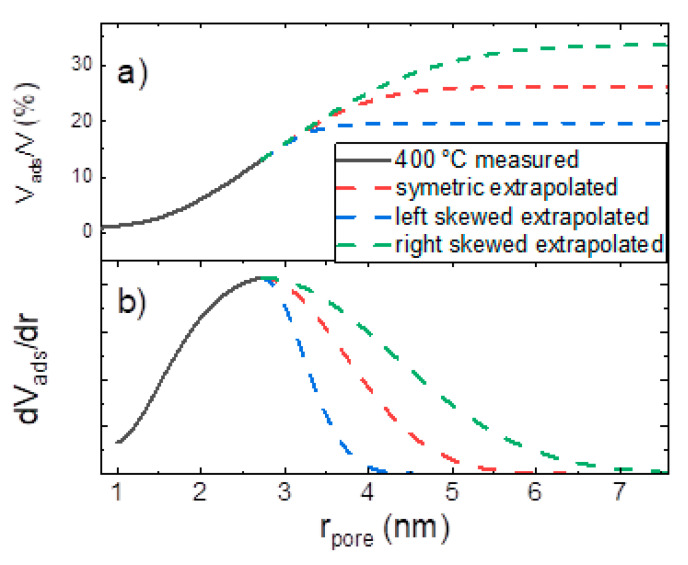
PSD error estimation for the sample calcinated at 400 °C: (**a**) adsorbed volume and (**b**) pore size distribution.

Regarding the total porosity value obtained from the extrapolation, an error estimation has been made. Therefore, unrealistic left and right skewed shapes of the PSD had been assumed (Figure 13b). Again, to gain the cumulative distribution (Figure 13a), the extrapolated PSD curves have been by integrated. The convergence values of the cumulative function obtained from the left skewed extrapolation yield the lower limit for the total porosity, namely 19.5%. Analogue, the right skewed extrapolation, yields 33.5% for the upper limit of the total porosity of the sample calcinated at 400 °C. When comparing this to the total porosity of calcinated DEZ/EG samples (see Table 2) one finds that the lowest limit for the porosity of the calcinated DEZ/EA/MA samples matches the measured value of the DEZ/EG samples. Yet, the according upper limit for the porosity of the calcinated DEZ/EA/MA samples is 14% higher than the measured value of the DEZ/EG samples. This clearly shows that in case of samples calcinated at 400 °C, it is highly likely to find an enhanced porosity for films deposited from the DEZ/EA/MA sequence compared to the ones obtained from the DEZ/EA sequence.

For the zinc-organic annealed to 400 °C, the overall open mesoporosity estimated is 25%, which is more than twice as much as in case of the ones heated to 500 and 600 °C, showing total open porosity values of 10 and 4%, respectively. Similar results have been reported for studies on samples deposited from DEZ and EG [2] (see Table 1), and were ascribed to the continuous, temperature enhanced crystallite growth between 340 and 600 °C. We also believe that in case of the samples deposited from DEZ, EA, and MA the ongoing crystallite growth lowers the porosity. However, these samples show a more complex transformation behavior upon heating (see Figure 12) compared to the samples obtained from simple AB-cycle MLD, as also indicated by the residual carbon content measured by FTIR for the samples calcinated up to 400 °C. More precise statements on the transformation mechanism and its influence on the porosity of the thin films would require elaborate in situ XRD measurements during the heating process.

Table 2 compares the porosity properties of ZnO after calcinating films deposited from both DEZ/EG and DEZ/EA/MA MLD cycles. Despite the lack of information on how the porosity of the three-step sequence MLD samples develops on a microscopic scale, it is evident that an increased organic content promotes the development of more but smaller pores upon heating to 400 °C, compared to the two-components zinc-organic films, for which, at the same temperature, an open porosity lower than 20% is reported [2]. The contrary applies to the samples heated to higher temperatures: The zinc-organic films obtained from DEZ, EA, and MA show lower porosity compared to the films obtained from DEZ and EG, for which the open porosity was >12% upon heating to 600 °C [2].

## 4. Conclusions

Zinc-organic layers were formed for the first time via a three-step MLD sequence using DEZ, EA, and MA. After initial degradation the zinc-organic layers were found to be stable in ambient air.

The measured saturation curves suggest a GPC of 4.4 ± 0.2 Å/cycle using the ideal deposition recipe DEZ/purge/EA/purge/MA/purge of 0.3/100/4 × 1.5/140/4 × 0.1/100 s. It is also worth mentioning that a GPC of 2.2 Å/cycle was found for a saturated two-step MLD process using only DEZ and EA.

With the aim of enhancing the porosity of the ZnO films obtained from calcinating the organic content, the zincone layers deposited via the three-step MLD sequence were heated up to a maximum temperature of 600 °C.

In situ SE measurements of the calcination process show a collapse of the metal-organic hybrid film between 300 and 370 °C assigned to the removal of the organic content of the film. Between 370 and 600 °C, the film thickness remains constant while the refractive index suggests an internal transformation of the film that we believe is caused by the formation of vacancies and a subsequent crystallization process at the cost of the film porosity [2]. The cooling behavior of the film’s refractive index indicates the presence of crystalline ZnO after calcination.

PSDs were obtained for samples heated to 400, 500, and 600 °C, yielding open porosity values of 25, 10, and 4% as well as pore radii expectation values of 2.8, 2, and 1.8 nm, respectively. Hence, compared to calcinated two-step MLD zincone samples obtained in a previous work we indeed find enhanced porosity for samples heated to 400 °C [2]. The FTIR showed a residual carbon content and a low crystallinity after the 400 °C temperature treatment, corroborating that pore formation and crystallization are competitive phenomena for the ZnO thin films. For higher calcination temperatures, the two-step MLD zincone samples show higher porosity.

## Figures and Tables

**Table 1:** Heating temperatures of the MLD system reported in Figure 2.

System Part	T °C	System Part	T °C
Reactor	60	DEZ line	42
Sample stage	60	EA & MA line	55
Exhaust line	60	EA & MA vessel	50

**Table 2:** Porosity and average pore radii of calcinated ZnO thin films obtained from DEZ/EG*2*- and DEZ/EA/MA-cycle MLD for different heating temperatures.* This value is estimated.

	DEZ/EG [2]		DEZ/EA/MA	
T (°C)	*r_pore_* (nm)	Porosity (%)	*r_pore_* (nm)	Porosity (%)
400	1.6	19.5	2.8	25 *
500	-		2	10
600	2.3	12.6	1.8	4

## Data Availability

Data sharing is not applicable to this article.
